# AliGROOVE – visualization of heterogeneous sequence divergence within multiple sequence alignments and detection of inflated branch support

**DOI:** 10.1186/1471-2105-15-294

**Published:** 2014-08-30

**Authors:** Patrick Kück, Sandra A Meid, Christian Groß, Johann W Wägele, Bernhard Misof

**Affiliations:** Zoologisches Forschungsmuseum A. Koenig, Adenauerallee 160-163, 53113 Bonn, Germany; University of Amsterdam, Amsterdam, Netherlands

**Keywords:** Software, Alignment quality, Sequence heterogeneity, Topological node support

## Abstract

**Background:**

Masking of multiple sequence alignment blocks has become a powerful method to enhance the tree-likeness of the underlying data. However, existing masking approaches are insensitive to heterogeneous sequence divergence which can mislead tree reconstructions. We present AliGROOVE, a new method based on a sliding window and a Monte Carlo resampling approach, that visualizes heterogeneous sequence divergence or alignment ambiguity related to single taxa or subsets of taxa within a multiple sequence alignment and tags suspicious branches on a given tree.

**Results:**

We used simulated multiple sequence alignments to show that the extent of alignment ambiguity in pairwise sequence comparison is correlated with the frequency of misplaced taxa in tree reconstructions. The approach implemented in AliGROOVE allows to detect nodes within a tree that are supported despite the absence of phylogenetic signal in the underlying multiple sequence alignment. We show that AliGROOVE equally well detects heterogeneous sequence divergence in a case study based on an empirical data set of mitochondrial DNA sequences of chelicerates.

**Conclusions:**

The AliGROOVE approach has the potential to identify single taxa or subsets of taxa which show predominantly randomized sequence similarity in comparison with other taxa in a multiple sequence alignment. It further allows to evaluate the reliability of node support in a novel way.

**Electronic supplementary material:**

The online version of this article (doi:10.1186/1471-2105-15-294) contains supplementary material, which is available to authorized users.

## Background

Alignment masking as a measure of reducing noise in sequence alignments is regularly applied in phylogenetics. The idea behind the concept of masking blocks of sequence alignments is the reduction of the unpredictable influence of substitution saturation and/or ambiguously aligned blocks of sequence alignments on subsequent tree reconstructions [1–8] by increasing the tree-likeness of the data. Simulations and analyses of alignment masking of empirical data corroborate the correctness of this idea. Currently, software packages mask complete blocks of multiple sequence alignments applying either arbitrarily chosen thresholds of sequence variability within alignment columns (e.g. software Gblocks [1, 2] and REAP [9]), or automatically adjusted thresholds depending on the input alignment (e.g. trimAl [4] and BMGE [6]), or applying a sliding window approach to identify blocks of predominantly high alignment ambiguity (ALISCORE [5, 7]). All methods exclude complete alignment blocks instead of sequence subsets thus masking also potentially valuable data for subsets of taxa.

Due to their design all masking methods are relatively insensitive to heterogeneous sequence divergence of single taxa. This is an important deficiency of masking methods, because heterogeneous sequence divergence can cause strong biases in tree reconstructions, for example long branch effects or the misplacement of rogue taxa. Therefore, a method which can visualize heterogeneous sequence divergence or alignment ambiguity related to single taxa or subsets of taxa would be a useful complement to currently used masking approaches. It offers the chance to identify taxa which are potentially misplaced in trees and reduce the tree-likeness of the data.

For this purpose, we developed AliGROOVE, a new tool to visualize the extent of sequence similarity and alignment ambiguity in pairwise sequence comparisons derived from a multiple sequence alignment. AliGROOVE can help to detect strongly derived sequences that have the potential to bias tree reconstructions and node support. We implemented an adaptation of the recently published ALISCORE masking algorithm [5, 7] which has been successfully tested in simulations and on empirical data [5, 7, 8]. Using a simple match/mismatch scoring for nucleotide data and a BLOSUM62 scoring matrix for amino acid data ALISCORE uses a Monte Carlo resampling within a sliding window to generate profiles of pairwise sequence similarity for all pairwise sequence comparisons. AliGROOVE summarizes site scores of these profiles normalized over the whole alignment length for each pairwise comparison. The obtained scoring values between sequences are translated into a similarity matrix and thus deliver information on the extent of taxonomically heterogeneous alignment ambiguity or sequence similarity within a multiple sequence alignment.

We used simulated data to investigate if our application of the algorithm is able to detect ambiguously aligned taxa or groups of taxa and if the obtained sequence similarity scores can be used to tag unreliable nodes. For that purpose we tested AliGROOVE on data sets with and without indel events whereby tests on data sets with indel events are performed on correct and on realigned data sets that deviate from the true alignment. Additionally, we applied AliGROOVE on an empirical data set comprising five mitochondrial genes of 53 chelicerate ingroup taxa and eight myriapod outgroup taxa. With both the simulated and empirical data sets we also tested the potential of the approach to illustrate heterogeneous tree-likeness among data blocks within an alignment.

### AliGROOVE Algorithm

#### Identification of sequence similarity/scoring

The algorithm of AliGROOVE is based on the scoring scheme of ALISCORE [5, 7] which compares pairs of amino acid/DNA sequences for random similarity within a sliding window. In short, first, the observed mismatch within the sliding window is scored. This mismatch score is then compared with mismatch scores of the same window size generated by permutations of character states within the sliding window and a predefined sequence neighborhood. If the observed score is better than 95% of the score of all generated permutations, it is considered non-random, otherwise indistinguishable from random similarity. Each position within the sliding window receives a positive sign if the observed score was significantly better than scores of random sequence similarity, or if not, a negative sign. The number of single signs for each alignment position corresponds to the size of the sliding window. For each position signs are summed up and normalized by the sliding window size. A profile of sequence similarity between two sequences will thus show sections in which these two sequences might show non-random similarity indicated by a positive sum of signs and sections of random similarity expressed by a negative sum of signs for each position. Now, for each profile the AliGROOVE algorithm calculates an arithmetic mean of profile signs over all sites excluding globally invariant sites within the alignment and records these values in a matrix for a given set of sequences. The entries in this similarity matrix express the average amount of non-random versus random similarity in pairwise comparisons and can thus illustrate heterogeneous signal in the data.

The algorithm is based on either match/mismatch scores for nucleotide sequences or on amino acid substitution matrices (BLOSUM62, PAM250, PAM500) to score amino acid matches/mismatches. This scoring regime turned out to be efficient in alignment masking [5, 7, 8, 10–18].

#### Identification of suspicious branches

AliGROOVE pairwise similarity scores can be used to tag potentially unreliable relationships in a pre-defined tree. Potentially unreliable relationships can be caused by extensive substitution saturation or extensive alignment ambiguity both causing long branches in a tree which can occurr in inner and terminal branches.

AliGROOVE tags terminal branches with the mean pairwise similarity score (*S*_*XY*_) between the terminal taxon and all other taxa. For example, the terminal branch of taxon A in a six taxon topology (taxa A to F), is tagged with *R*_*A*_ defined as:
1

To tag internal nodes, AliGROOVE calculates the mean similarity score from all pairwise comparisons across this node. The tagging of the internal nodes follows the hierarchy given by a topology and ends at the most central internal branch. Following a guiding topology effectively reduces the number of splits to be analyzed to the ones which are of special interest. This reduction of the complexity of analyses makes the approach computationally efficient. For example, to tag the internal branch separating taxa A and B from the remaining taxa (taxa C to F), AliGROOVE calculates *R*_*AB*|*CDEF*_defined as:
2

The calculation of the mean pairwise similarity score treats all pairwise comparisons as independent replicates. This assumption is not justified in every case. For example, taxa C and E might be closely related and *S*_*AC*_ and *S*_*AE*_ do not represent fully independent replicates.

## Results

### Testing the performance with simulated data (Setup A & B)

**Figure 1:**
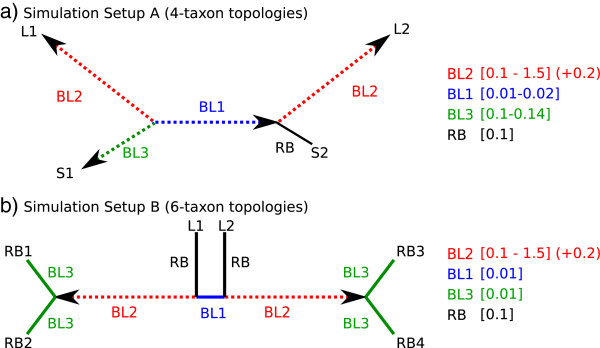
**Simulation setup A and B.** Two sets of nucleotide and amino acid data using **a)** 4-taxon (setup A) and **b)** 6-taxon topologies (setup B). Setup A contained two elongated, non directly related terminal branches (*B* *L* 2 = 0.1,0.3,0.5,0.7,0.9,1.1,1.3,1.5) under three different branch length conditions of remaining short branches (*B* *L* 3 = 0.1,0.12,0.14 and *R* *B* = 0.1) and two different lengths of the very short internal branch (*B* *L* 1 = 0.01,0.02). Setup B contained two elongated internal branches (*B* *L* 2 = 0.1,0.3,0.5,0.7,0.9,1.1,1.3,1.5), separated by a short internal branch (*B* *L* 1 = 0.01) while terminal branches are kept constant (*B* *L* 3 = 0.01 and *R* *B* = 0.1).

**Figure 2:**
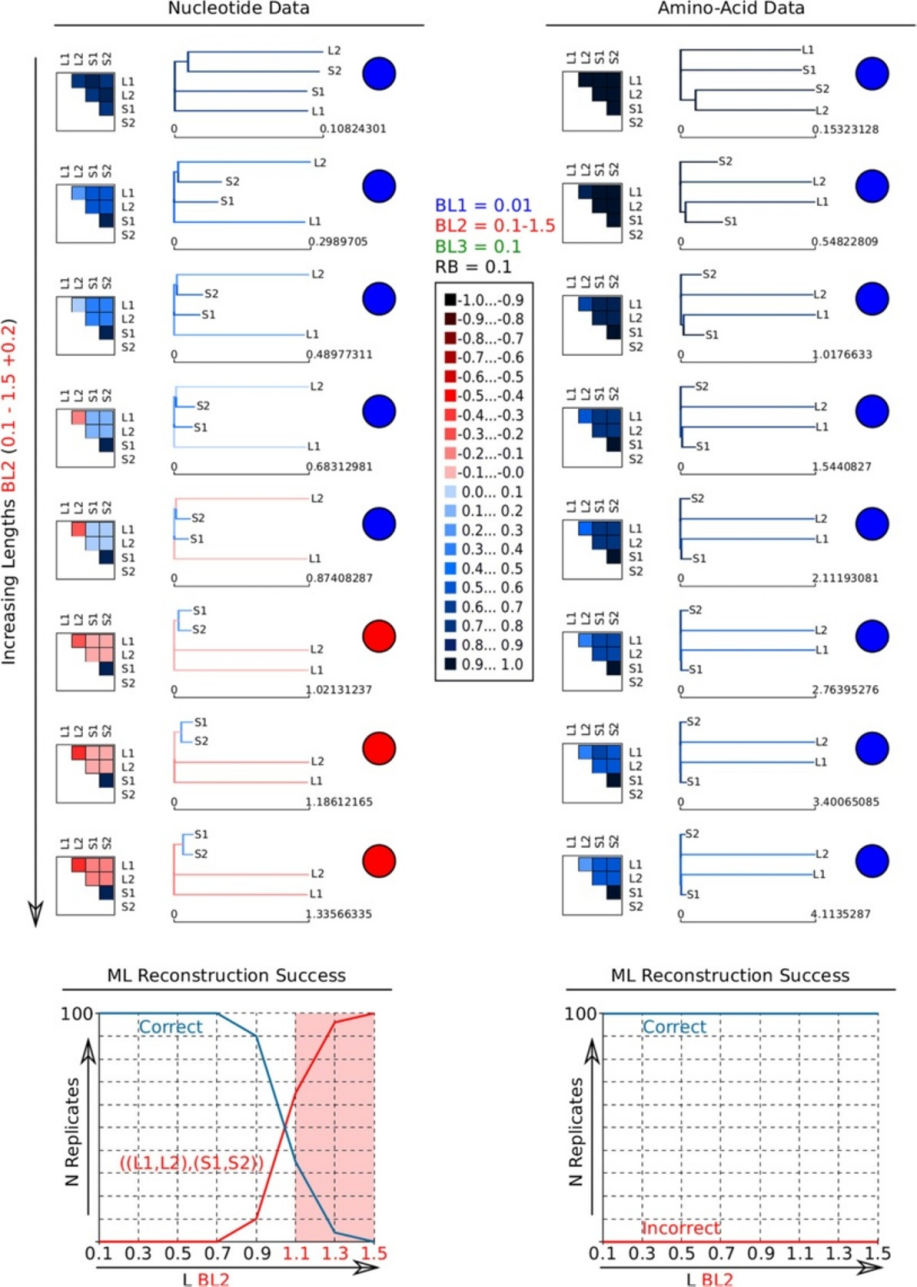
**AliGROOVE example results of 4-taxon simulation tests (simulation setup A).** AliGROOVE similarity scores and identified branch reliability of best Maximum Likelihood (ML) topologies obtained for different branch elongations of two non-directly related terminal branches (L1, L2) (Figure 1a) considering nucleotide and amino acid data (sequence length: 250,000 bp). The two graphs below show the reconstruction success in relation to the length of long branches (BL2). Note that amino acid sequences are more reliable. Colour coded similarity score ranges are shown in the center. Lacking reliability of internal branches (red internal branch) is observed for incorrect ML topologies predominating in 100 data replicates conducted for each length of BL2. Boxes with coloured squares show scores for pairwise sequence comparisons. In the corresponding topologies unreliable branches are shown in red. Circles indicate whether the topologies are correct (blue) or wrong (red). All results of 4-taxon simulations are given as Additional files 1 and 2.

**Figure 3:**
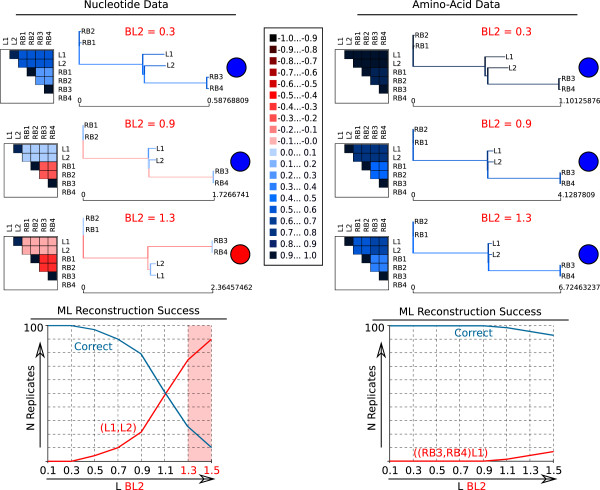
**AliGROOVE example results of 6-taxon simulation tests (simulation setup B).** Simulation setup B (Figure 1b) using six taxa (sequence length: 50,000 bp). The overall ML reconstruction success corresponds to pairwise similarity scores obtained between L1,L2 and remaining taxa (RB1-RB4) and decreases with a more frequent incorrect grouping of (L1,L2) with increasing lengths of BL2 (from dark blue to red). Colour coded similarity score ranges are shown in the center. Lacking reliability of internal branches connecting L1 and L2 scores negatively (red internal branch) in cases where the corresponding ML topologies are incorrect (red circles). All results of 6-taxon simulations are given as Additional file 3.

We simulated nucleotide and amino acid sequence alignments under two different topological conditions (Figure 1). Our first setup represents 4-taxon trees (setup A) containing long terminal branches BL2 (Figure 1a). This setup has been selected to reduce the complexity of phenomena and to demonstrate the ability of AliGROOVE to identify heterogeneous sequences which can cause long branch attraction of terminal branches. Our second simulation setup consists of 6-taxon trees (setup B) containing long internal branches BL2 (Figure 1b). The frequencies of correct and incorrect Maximum Likelihood tree reconstructions using nearly correct model assumptions (using four rate categories instead of a continuous *Γ* distribution) were recorded (Figures 2, 3). To simulate large-scale phylogenetic analyses based on concatenated supermatrices, setup A comprises alignment lengths of 250,000 sites, while setup B has alignment lengths of 50,000 sites. The shorter sequence lengths of setup B have been chosen to reduce computational time of our 6-taxon analyses.

In setup A (Figure 1a), we simulated data with increasing terminal branch lengths of two unrelated taxa. For increasing branch length conditions the similarity scores between sister taxa correlate with tree reconstruction success ((L1,S1) & (L2,S2) in Figure 2). The mean similarity scores for internal branches are as well correlated with the tree reconstruction success. Negative mean similarity scores are directly correlated with tree reconstruction errors. Using AliGROOVE with the tree tagging option to project the observed pairwise sequence similarity scores on a provided guiding tree, the internal branch connecting two groups of taxa is tagged as suspicious (red colored) when the observed similarity score of this branch receives a negative value. A complete overview of all results is given in the Additional files 1 and 2.

In setup B, we simulated multiple sequence alignments with two internal nodes using 6-taxon trees (Figure 1b). The results lead again to the conclusion, that there is a correlation between the similarity score of the two long internal branches and tree reconstructions, which were predominantly incorrect in case of negative scores (*BL*2≥ 1.1) (Figure 3). For example, in setup B taxa L1 and L2 are connected to the remaining taxa via two long internal branches. With increasing internal branch lengths taxa L1 and L2 occur more often as sister group instead of being paraphyletic in relation to remaining taxa. In this case, taxa L1 and L2 will share character states which have been lost in other taxa inducing a wrong sistergroup relationship based on plesiomorphies. By using the ALIGROOVE approach with the tree tagging option, correctly reconstructed short internal branches assigning taxa L1 and L2 as paraphyletic groups have been tagged as non-suspicious, whereas incorrectly resolved short internal branches have been identified as suspicious. Whenever branch lengths are balanced, tree reconstructions have been continuously successful, which is also reflected by the similarity scores obtained for the alignments of these topologies (see Figure 3). All AliGROOVE results of the 6-taxon setup are shown in the Additional file 3.

### Testing the performance on simulated data setup C

**Figure 4:**
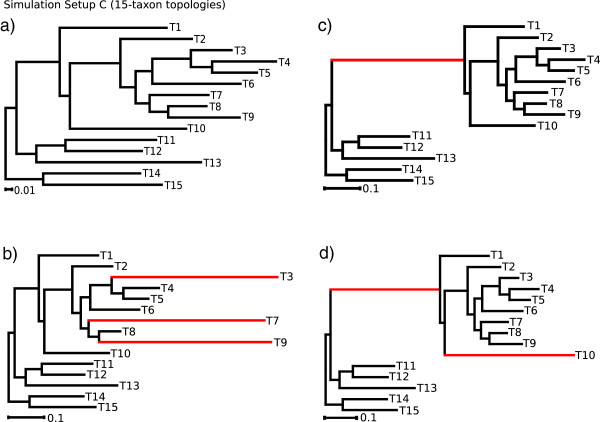
**Simulation setup C.** Nucleotide data simulation with and without indel events based on four different branch length conditions of a 15-taxon topology. Elongated branches of topology C2 **(b)**, C3 **(c)**, and C4 **(d)** in comparison to topology C1 **(a)** are highlighted red.

**Figure 5:**
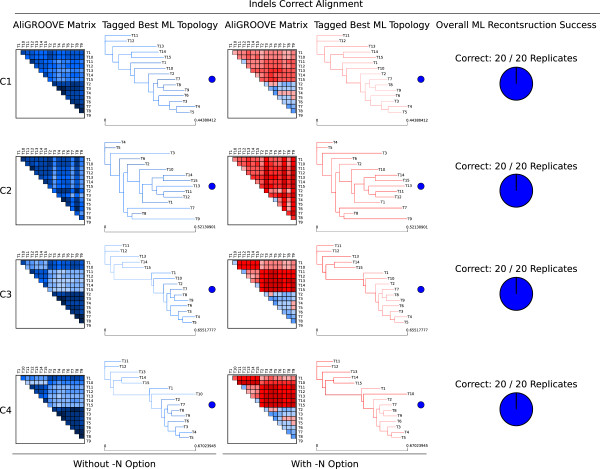
**AliGROOVE example results of correctly aligned 15-taxon simulation tests (simulation setup C).** AliGROOVE similarity scores and identified branch reliability of best Maximum Likelihood (ML) topologies as well as the obtained overall ML reconstruction success for four different branch length conditions of a 15-taxon topology (Figure 4) under the GTR model of sequence evolution used for data simulation and tree reconstruction. Correctly aligned data sets have been simulated with indel events and analysed by AliGROOVE without -N option (indel events are treated as fifth character state) or with -N option (indel events treated as ambiguous characters).

**Figure 6:**
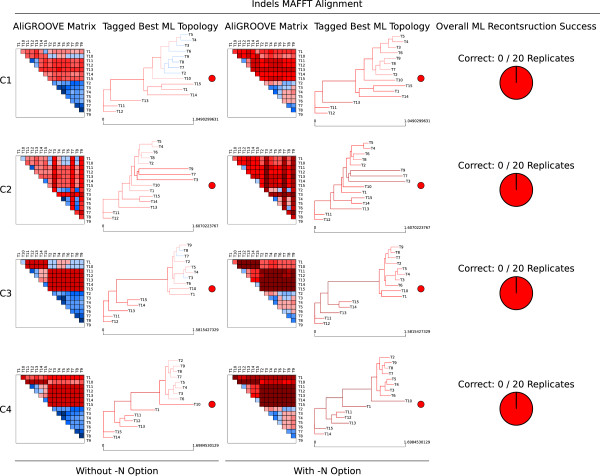
**AliGROOVE example results of MAFFT realigned 15-taxon simulation tests (simulation setup C).** AliGROOVE similarity scores and identified branch reliability of best Maximum Likelihood (ML) topologies as well as the obtained overall ML reconstruction success for four different branch length conditions of a 15-taxon topology (Figure 4) under the GTR model of sequence evolution used for data simulation and tree reconstruction. Realigned (MAFFT) data sets have been simulated with indel events and analysed by AliGROOVE without -N option (indel events are treated as fifth character state) or with -N option (indel events treated as ambiguous characters).

In setup C, we simulated data sets with and without indel events under four different branch length conditions of a 15-taxon topology (Figure 4) and two different models of sequence evolution (Jukes-Cantor and General Time Reversible model). Both models of sequence evolution used for data simulations led to similar AliGROOVE results (Additional file 4). Pairwise sequence comparisons of data sets simulated without indel events receive positive similarity scores in all four 15-taxon topologies and reconstructed trees are always correct (Additional file 4). Correctly aligned data sets simulated with indel events receive positive similarity scores when indel events are treated as fifth character (Figure 5). Strongly divergent sequences receive negative similarity scores if indel events are treated as ambiguous characters. The high overall reconstruction success obtained from ML analyses correlates with the AliGROOVE results obtained with indels as fifth character (Figure 5, Additional file 4). These data sets realigned receive negative similarity scores independently of the chosen indel scoring (Figure 6), whereas similarity scores decrease under both settings compared to scorings inferred from correct multiple sequence alignments (Figures 5, 6). For all simulated branch length conditions, the incorrect placement of long internal and terminal branches could be identified successfully in realigned data sets with both scoring options (Figure 6, Additional file 4).

### Testing the performance on simulated data setup D

**Figure 7:**
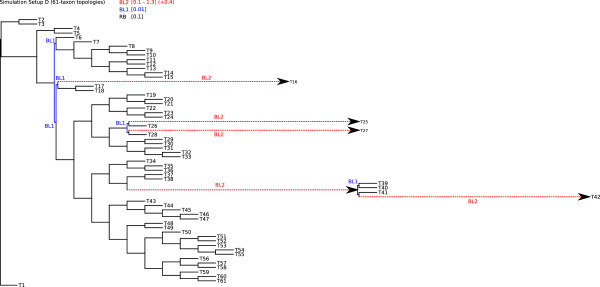
**Simulation setup D.** 61-taxon setup used for simulation of nucleotide and amino acid gene partitions. Alignment lengths of single gene partitions were set to 500, 1000, 1500, 2000, and 2500 character state positions. To simulate different substitution rates, internal and terminal branches were stepwise increased for each gene partition length (highlighted red), ranging from 0.1 to 1.3 (*B* *L* 2 = 0.1,0.5,0.9,1.3). Internal branches in close vicinity to elongated branches are kept very short (*B* *L* 1 = 0.01). All remaining branches are kept equally long (*R* *B* = 0.1).

**Figure 8:**
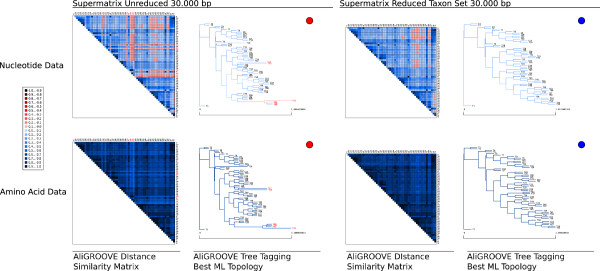
**AliGROOVE results of concatenated gene analyses on unreduced and reduced 61-taxon simulation setup D.** AliGROOVE similarity score distance matrices and associated ML topologies of the original supermatrix and the taxon reduced supermatrix, including all 20 gene partitions. The darker blue the colour coded similarity scores in AliGROOVE matrices, the higher the non-randomized accordancy between pairwise sequence comparisons. Red indicates the opposite. Tagged branch reliability of associated best ML topologies is given next to each matrix. Correct reconstructed topologies are pointed blue, incorrect trees red. Names of incorrectly resolved sequences are highlighted red.

In setup D, we simulated nucleotide and amino acid sequence data sets for a 61-taxon tree with data block sizes of 500, 1000, 1500, 2000, and 2500 sites under four different branch length conditions (BL2 = 0.1, 0.5, 0.9, 1.3) (Figure 7). Tree reconstruction analyses were performed with correct site rate heterogeneity and proportion of invariant sites model parameters. Maximum Likelihood tree reconstructions were correct for unreduced (all sequences) nucleotide and amino acid sequence data sets with branch lengths of BL2 = 0.1 and alignment lengths above 500 sites. For all other setups, Maximum Likelihood failed to find the correct tree for the nucleotide sequence data sets and delivered correct trees for amino acid sequence data sets only in case of data blocks larger than 2000 sites and branch length BL2 less or equal 0.9 (Additional file 5, Additional file 6). At least one of the five long branches was always misplaced in incorrect trees (Figure 8, Additional file 5, Additional file 6). Thus, the seven highly divergent sequences (T16, T25, T27, T39, T40, T41, and T42) were problematic in nucleotide and most amino acid sequence data sets.

With the AliGROOVE algorithm, the highly divergent seven nucleotide sequences did not consistently cause negative scores in all pairwise sequence comparisons if branch lengths of BL2 were set to 0.5, but got almost always negative scores if BL2 were set to ≥ 0.9 and data blocks to >1000 sites (Additional file 5). With amino acid datsets, the seven highly divergent sequences got only positive scores in all pairwise sequence comparisons, independently of the tree reconstruction success (Additional file 6).

The tree tagging algorithm tagged all highly divergent nucleotide sequences and associated long branches as unreliable for branch lengths BL2 ≥ 0.9, and tagged all incorrectly placed nucleotide sequences and associated long branches as unreliable if sequence length of nucleotide data blocks was set to 2500 sites and branch lengths BL2 = 0.5. In case of shorter data blocks and branch lengths set to BL2 = 0.5, tagging was less consistently correct (Additional file 5). For amino acid datsets, non of the seven highly divergent sequences and associated long branches were tagged as unreliable.

These results also apply to the concatenated nucleotide and amino acid supermatrix data sets which consist of all data blocks. The AliGROOVE pairwise distance similarity matrix of the concatenated nucleotide supermatrix shows the seven highly divergent sequences mostly red colored, however despite being misplaced on the tree, the branches associated with this seven highly divergent sequences are not consistently tagged as suspicious (Figure 8). With the amino acid supermatrix, the highly divergent sequences are not highlighted in the distance matrix and branches associated with these sequences are not tagged as suspicious, despite being wrong. For both nucleotide and amino acid supermatrices the exclusion of the seven divergent sequences led to correct topologies (Additional file 7).

In general, the AliGROOVE tagging algorithm is optimistic concerning the reliability of branching patterns and never tags a branch as unreliable if in fact correct.

### Testing the performance with empirical mitochondrial data

**Figure 9:**
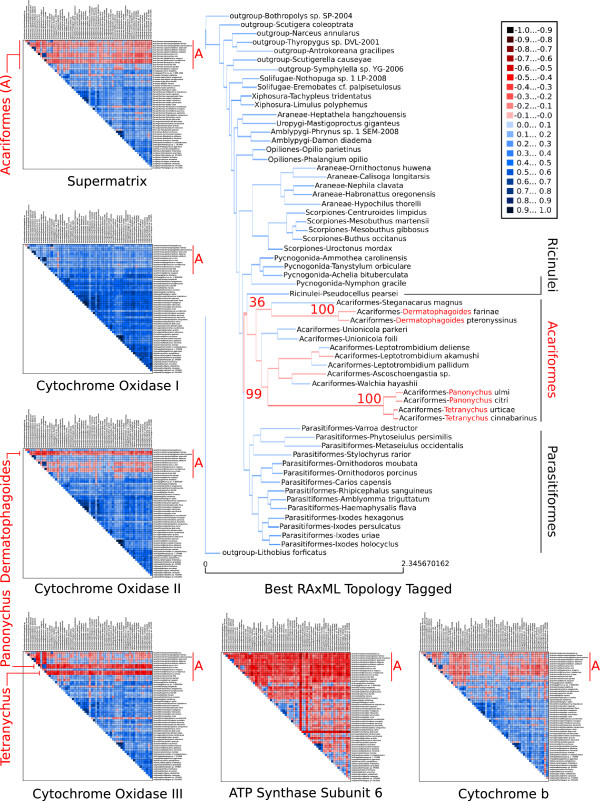
**AliGROOVE results of concatenated and single gene analyses on mitochondrial data of Chelicerata taxon groups.** Except of Cytochrome Oxidase I (mainly positive pairwise similarity scores) and ATP6 (mainly negative pairwise similarity scores), AliGROOVE identified mainly negative similarity scores in pairwise comparisons whenever sequences of Acariformes (A) are involved. Although subgroups within Acariformes get a higher bootstrap support in the best ML tree using the supermatrix data (shown here), they are tagged with AliGROOVE as unreliable. The misleading information accumulates for these taxa especially in the COII and COIII sequences, while ATP6 is generally to noisy. In the concatenated data set (“supermatrix”) the misleading patterns are still dominant.

We used mitochondrial DNA sequence data downloaded from the NCBI genome data base for 53 chelicerate ingroup taxa and eight myriapod outgroup taxa. It is known that among chelicerates the Acari (mites, ticks) are problematic [19, 20]. AliGROOVE analyses of the concatenated supermatrix file and of gene partitions showed that pairwise sequence comparisons involving mite sequences received negative scores while pairwise comparisons between other sequences achieved mainly positive scores (Figure 9). Among all gene partitions, only the Cytochrome Oxidase I (COI) DNA sequence alignment shows positive similarity scores for nearly all taxon comparisons. While nearly all pairwise sequence comparisons of the ATP Synthase Subunit 6 (ATP6) yielded negative similarity scores, impacts of random sequence similarity and alignment ambiguity vary for mite subgroups in Cytochrome b (Cytb), Cytochrome Oxidase II (COII), and Cytochrome Oxidase III (COIII). For Cytb, mite sequences are not highly divergent whereas specific mite subgroups appear strongly misaligned in COII (*Dermatophagoidae*) and COIII (*Panonychus* & *Tetranychus*). These three mite subgroups are also scored constantly negative in pairwise comparisons of the concatenated supermatrix. Nevertheless, the phylogenetic position of *Dermatophagoidae*, *Panonychus* and *Tetranychus* receives high bootstrap support in the tree reconstruction based on the concatenated supermatrix. The supermatrix sister group relationship of Acariformes and Ricinulei with a bootstrap support of 36 was as expected tagged as unreliable (red colored) with AliGROOVE. However, the supermatrix clade ((Ricinulei, Acariformes), Parasitiformes) that received a bootstrap support of 99 was tagged as unreliable as well (Figure 9).

## Discussion

It has been shown that traditional masking of entire sequence alignment blocks can improve the signal-to-noise ratio or tree-likeness in sequence alignments. Here, we show that the sliding window approach as it is used in ALISCORE [5, 7] can be modified to identify single taxa or subsets of taxa which show predominantly randomized sequence similarity in comparison with other taxa (Figure 9). Masking of these taxa can also improve the signal-to-noise ratio in sequence alignments. The approach implemented in AliGROOVE can be used to test the reliabilities of reconstructed topologies and to identify unreliable node support in a user specified tree (Figures 2, 3,5, 6, 8, 9, Additional files 1, 2, 3, 4, 5, 6). This possibility offers a convenient way of studying node support in a given tree and multiple sequence alignment complementary to conventional bootstrap analyses. The identification of taxonomic subsets offers the possibility to mask only taxonomic sub-blocks of multiple sequence alignments that clearly contain the least signal due to alignment ambiguity, sequence saturation or excessive divergence.

Results of the analyses of simulated nucleotide data sets with indel events and/or missing data (coded as gaps) and correct sequence alignment showed that the AliGROOVE approach correctly identified excessively divergent sequences with treating indels as fifth character state (Figure 5, Additional file 4). After realigning these data, the difference between treating indels as fifth or ambiguous character state vanished. This may be explained by misplaced indels during the process of realignment which should be better treated as ambiguous character states. For empirical data, in particular indel-rich data in which we cannot discriminate between misplaced and correctly placed indels, this result implies that indels should be treated as ambiguous character state or completely removed from phylogenetic analyses [2, 4, 21].

The results concerning simulation setup D merit additional discussions. In these analyses, branch length differences between clades have been pushed to the extreme. With nucleotide sequences, the AliGROOVE algorithm correctly tagged misplaced branches if BL2 ≥ 0.9. With amino acid data even these long branches were never tagged as unreliable despite being incorrectly placed. Apparently, detectable substitutional saturation accumulated only if branch lengths BL2 were ≥ 0.9, and extremely short internal BL1 =0.01 were insufficient to accumulate any signal. This phenomenon was pronounced for amino acid data. The extremely short internal branch lengths of BL1 =0.01 can be interpreted as hard polytomies, for which tree reconstructions cannot deliver correct results. However, the frequency of hard polytomies limiting the application of the AliGROOVE algorithm in empirical data is currently unknown.

The mitochondrial DNA sequence data set of chelicerates shows strong heterogeneity of sequence divergence as indicated in the similarity matrix (Figure 9). Specimens of Acariformes display mostly random similarity to all other sequences. This observation implies that Acariformes cannot be robustly placed in the tree or are potentially misplaced despite robust bootstrap support. This is exactly what we see in the tree reconstruction using the concatenated supermatrix data set, as Acariformes are sister group to Ricinulei and form together with Parasitiformes the sister group to Pycnogonidae. This grouping which is considered implausible by many specialists [19, 20, 22, 23] gets a high bootstrap support. The questionable sister group relationship between Ricinulei and Acariformes has been identified with AliGROOVE and is tagged as suspicious in the topology inferred from the supermatrix. The AliGROOVE algorithm clearly identified the most problematic sequences and gene partitions in the data set and demonstrates its usability with this data.

## Conclusions

**Figure 10:**
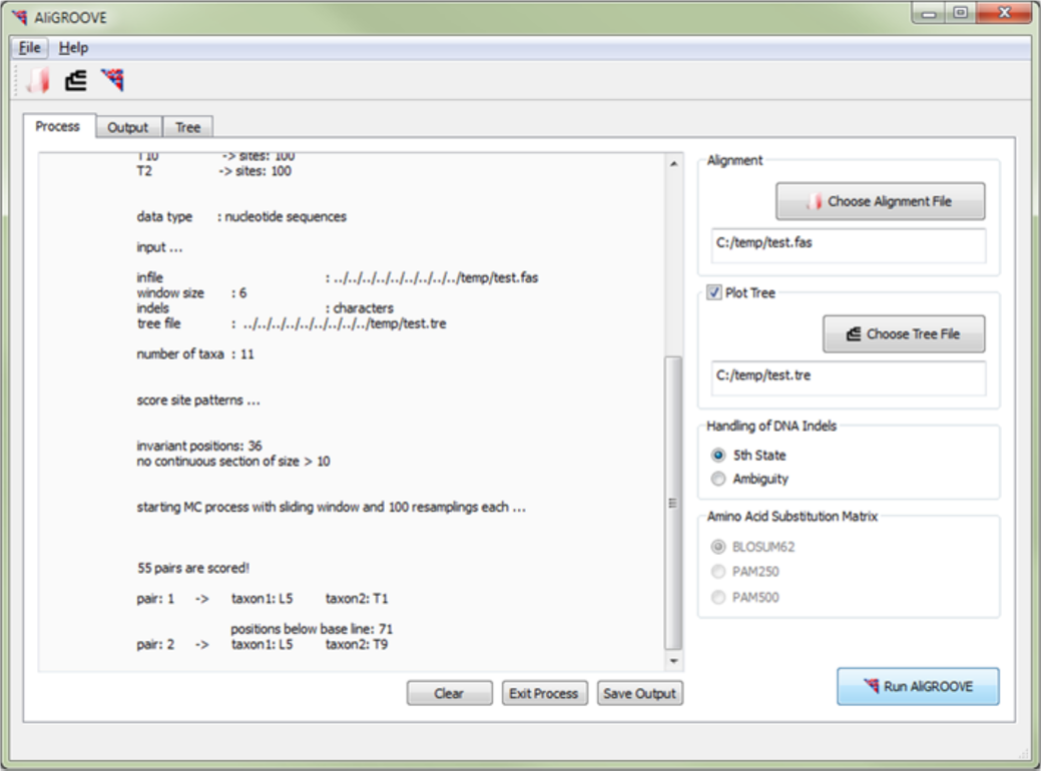
**Graphical user interface (GUI) version of AliGROOVE.** Overview of the AliGROOVE process window.

The analyses of the simulated and the empirical data show that the sliding window approach identifies relevant sources of reconstruction error. Therefore, we suggest our method as an important complement to all character based masking approaches in phylogenetics. It offers the possibility to exclude taxa or gene partitions based on a formal argument instead of excluding taxa based exclusively on the evaluation of branch lengths. The exclusion or exchange of conflicting sequences and/or gene partitions improves the signal-to-noise ratio of the alignment and, as a consequence of this, can lead to less biased, more realistic trees. The simple usage of the AliGROOVE program via graphical user interface (Figure 10) facilitates the identification of potentially problematic taxa or gene partitions for users which feel uncomfortable with command line based software while the alternatively available command line version of AliGROOVE can be easily integrated into automated analysis pipelines. AliGROOVE has no maximum limit in taxon number or sequence length.

## Material and methods

### Simulated data setup A & B

To test the efficiency of AliGROOVE we designed two sets of nucleotide and amino acid sequence data using 4-taxon and 6-taxon trees (Figure 1). The topology of the 4-taxon setup (setup A, Figure 1a) contained two long branches of unrelated taxa (with branch lengths *BL* 2 = 0.1,0.3,0.5,0.7,0.9,1.1,1.3,1.5) under three different branch length conditions for the other two short terminal branches (*BL* 3 = 0.1,0.12,0.14 and *RB* = 0.1) and two different lengths of the short internal branch (*BL* 1 = 0.01,0.02). The 6-taxon setup (setup B, Figure 1b) contained two long internal branches (*BL* 2 = 0.1,0.3,0.5,0.7,0.9,1.1,1.3,1.5), separated by a short internal branch (*BL* 1 = 0.01) while the lengths of terminal branches are kept constant (*BL* 3 = 0.01 and *RB* = 0.1). For both test setups, 100 alignments were generated for each step of *BL* 2 branch elongation. Sequence length of each alignment of setup A was set to 250,000 character state positions and for setup B to 50,000 character state positions to reduce the calculation time. All alignments were generated with INDELible v.1.03 [24]. In order to simulate nucleotide sequence data we used the Jukes-Cantor model (JC) of sequence evolution and for amino acid sequence data the BLOSUM62 substitution model. All data were simulated with among site rate variation (ASRV), using a mixed-distribution model with a shape parameter *α* = 1.0, and a proportion of invariant sites *ρ*_*inv*_= 0.3. ASRV was modelled using a continuous *Γ*-rate distribution while indel events were not simulated.

Trees of simulated data were inferred with PhyML_3.0_linux64 [25, 26]. We analyzed the data with a mixed-distribution model (JC+ *Γ* + I) and correct parameter values (*α* = 1.0, *ρ*_*inv*_= 0.3), except for the categorization of the gamma distribution. The number of relative substitution rate categories was set to four (c = 4) and tree topologies and branch lengths were optimized. Maximum Likelihood analyses were performed and evaluated with a Perl pipeline. For each branch length-combination, we generated 100 data replicates and recorded the frequencies of correct and incorrect tree reconstructions using correct alignments and nearly correct substitution models (Figures 2, 3, Additional files 1, 2, 3).

### Simulated data setup C

To test the efficiency of AliGROOVE when sequences contain gaps and missing data we simulated nucleotide sequence data sets for four different 15-taxon topologies (Figure 4). The -N option of AliGROOVE allows to toggle between scoring gaps as fifth character state or as ambiguity. The efficiency of AliGROOVE with and without the usage of the -N option was tested on correct alignments (Figure 5) and on realigned data sets using MAFFT [27, 28] under default values (Figure 6). Additionally, alignments were also simulated without indel events under otherwise identical parameter settings. Topologies differed only in branch lengths. While topology C1 (Figure 4a) consisted of more or less well balanced branch lengths, three terminal branches (Taxon T3, T7, T9) have been strongly increased in topology C2 (Figure 4b). One internal branch separating taxa T1 to T10 from remaining taxa has been strongly increased in topology C3 (Figure 4c), and one internal branch separating taxa T1 to T10 from remaining taxa as well as an addtional terminal branch (taxon T10) has been strongly increased in topology C4 (Figure 4d). Alignment lengths of simulation setup C were set to 50,000 sites. All data were simulated with ASRV, using a mixed-distribution model with a shape parameter *α*=0.5, and a proportion of invariant sites *ρ*_*inv*_= 0.1. ASRV was modeled using a continuous *Γ*-rate distribution while indel events were simulated using a Lavalette Distribution where the maximum indel length was set to 20. Insertion and deletion rate were both set to 0.2. Single state frequencies of GTR simulations were set to *T* = 0.35, *C* = 0.15, *A* = 0.35, *G* = 0.15.

Trees of simulated data were inferred with PhyML_3.0_linux64 [25, 26] using either the JC or GTR model of sequence evolution (depending on the substitution model used for data simulations) with a mixed-distribution model by estimating the *α* shape parameter and the proportion of invariant sites. The number of gamma shape rate categories was set to four (c = 4) and tree topologies and branch lengths were optimized. Maximum Likelihood analyses were performed and evaluated with a Perl pipeline. For each topology and AliGROOVE setting, we generated 20 data replicates and recorded the frequencies of correct and incorrect tree reconstructions (Figures 5, 6, Additional file 4).

### Simulated data setup D

To test the efficiency of AliGROOVE on large data sets and more realistic data block lengths, we simulated five different data block lengths of nucleotide and amino acid sequence data for a 61-taxon topology under four different internal and terminal branch length conditions (Figure 7). Alignment lengths of single data blocks were set to 500, 1000, 1500, 2000, and 2500 sites. To simulate different substitution rates for specific branches we stepwise increased single internal and terminal branches for data block length from 0.1 to 1.3 (*BL* 2 = 0.1,0.5,0.9,1.3). To increase rate heterogeneity between long branches and nearest-neighbour branches we kept internal branches very short (*BL* 1= 0.01). All remaining branches are kept at *RB* = 0.1. Our simulation setup lead to a total number of 20 gene partitions with each alignment length of data blocks being represented four times, each time with another substitution rate for specific taxa due to increased branch lengths of the data underlying topology.

Like in simulation setup A and B we simulated all data with ASRV, using a mixed-distribution model with a shape parameter *α* = 1.0, and a proportion of invariant sites *ρ*_*inv*_= 0.3. ASRV was modeled using a continuous *Γ*-rate distribution. Indel events were not simulated. In order to simulate nucleotide sequence data we used the Jukes-Cantor model (JC) of sequence evolution and the BLOSUM62 substitution model for amino acid sequence evolution. For sequence concatenation we used FASconCAT v1.0 [29].

Trees of simulated data were again reconstructed with PhyML_3.0_linux64 [25, 26] using the JC of sequence evolution (JC+ *Γ* + I) with correct rate heterogeneity and invariant site proportion parameters (*α* = 1.0, *ρ*_*inv*_= 0.3). The number of gamma shape rate categories was set to four (c = 4). All Maximum Likelihood analyses were performed and evaluated with a Perl pipeline.

AliGROOVE was tested on complete as well as reduced data blocks and supermatrices. Reduced sequence blocks and supermatrices were used to test the overall quality improvement of given data and associated trees after removing sequences which have been identifed as potentially unreliable in the majority of the AliGROOVE analyses (Additional files 5, 6, 7, 8).

### Empirical data

We used AliGROOVE without the -N option (indels coded as fifth character state) on a concatenated super alignment (5082 character state positions) as well as on corresponding single gene data sets of five mitochondrial genes (Atp6 ↪ 696 character state positions, COI ↪ 1575 character state positions, COII ↪ 783 character state positions, COIII ↪ 861 character state positions, and Cytb ↪ 1167 character state positions) downloaded from the NCBI genome data base for 53 chelicerate ingroup taxa and eight myriapod outgroup taxa. Single mitochondrial genes were aligned with ClustalW [30] and concatenated with FASconCAT [29]. The best ML topology of the mitochondrial data set was estimated using RAxML_7.2.2 [31] and the GTR+ *Γ* model. Single node support has been evaluated by performing 1000 bootstrap replicates (Figure 9).

### Computation time

Time complexity of AliGROOVE is given by:
3

M means the sequence length of a given alignment, N the total number of aligned taxon sequences. For example, the AliGROOVE computation time of a single 4-taxon alignment with sequence lengths of 250.000 character states took 809 seconds using a GenuineIntel(R) Core(TM) i7, 2.60GHz processor. The computation time of a 64-taxon data set with an alignment length of 2500 characters, conducting 1830 pairwise sequence analyses, took 2578 seconds.

### Implementation of AliGROOVE

AliGROOVE is implemented in Perl and runs on Linux, Mac OS, and Windows operating systems. It can be used via command line or graphical user interface (GUI). The GUI of AliGROOVE (Figure 10) is based on Qt, a cross-platform application and GUI framework in C ^++^.

## Availability of supporting data and requirements

-  
**Project name:** AliGROOVE – visualization of heterogeneous sequence divergence within multiple sequence alignments and detection of inflated branch support
-  
**Project home page:**http://zfmk.de/web/Forschung/Abteilungen/AG_Wgele/Software/AliGROOVE/index.en.html
-  
**Operating system(s):** Platform independent
-  
**Programming language:** Perl
-  
**Other requirements:** Perl 5.0 or higher
-  
**License:** GNU GPL version 2
-  
**Any restrictions to use by non-academics:** No restrictions

## Electronic supplementary material

Additional file 1: **Complete results of 4-taxon simulations based on nucleotide data.** Graphical result plots of all AliGROOVE analyses performed for nucleotide data based on 4-taxon topologies. The pdf document can be opened with pdf readers like AdobeAcrobatReader, Xpdf, or DocumentViewer. (PDF 67 KB)

Additional file 2: **Complete results of 4-taxon simulations based on amino acid data.** Graphical result plots of all AliGROOVE analyses performed for amino acid data based on 4-taxon topologies. The pdf document can be opened with pdf readers like AdobeAcrobatReader, Xpdf, or DocumentViewer. (PDF 65 KB)

Additional file 3: **Complete results of 6-taxon simulations.** Graphical result plots of all AliGROOVE analyses performed for nucleotide and amino acid data based on 6-taxon topologies. The pdf document can be opened with pdf readers like AdobeAcrobatReader, Xpdf, or DocumentViewer. (PDF 31 KB)

Additional file 4: **Complete results of 15-taxon simulations.** Graphical result plots of all AliGROOVE analyses performed for complete and indel events included nucleotide data based on 15-taxon topologies. The pdf document can be opened with pdf readers like AdobeAcrobatReader, Xpdf, or DocumentViewer. (PDF 251 KB)

Additional file 5: **Complete results of unreduced 61-taxon gene partitions simulations based on nucleotide data.** Graphical result plots of all AliGROOVE analyses performed for unreduced nucleotide gene partitions based on 61-taxon topologies. The pdf document can be opened with pdf readers like AdobeAcrobatReader, Xpdf, or DocumentViewer. (PDF 461 KB)

Additional file 6: **Complete results of unreduced 61-taxon gene partitions simulations based on amino acid data.** Graphical result plots of all AliGROOVE analyses performed for unreduced amino acid gene partitions based on 61-taxon topologies. The pdf document can be opened with pdf readers like AdobeAcrobatReader, Xpdf, or DocumentViewer. (PDF 448 KB)

Additional file 7: **Complete results of taxon reduced gene partitions based on 61-taxon nucleotide data simulations.** Graphical result plots of all AliGROOVE analyses performed for taxon reduced nucleotide gene partitions based on 61-taxon topologies. The pdf document can be opened with pdf readers like AdobeAcrobatReader, Xpdf, or DocumentViewer. (PDF 296 KB)

Additional file 8: **Complete results of taxon reduced gene partitions based on 61-taxon amino acid simulations.** Graphical result plots of all AliGROOVE analyses performed for taxon reduced amino acid partitions based on 61-taxon topologies. The pdf document can be opened with pdf readers like AdobeAcrobatReader, Xpdf, or DocumentViewer. (PDF 288 KB)
