# Supplementary material for: AliGROOVE – visualization of heterogeneous sequence divergence within multiple sequence alignments and detection of inflated branch support
Source: BMC Bioinformatics. 2014 Aug 30;15(1):294. doi: 10.1186/1471-2105-15-294 (PMC4167143; doi:10.1186/1471-2105-15-294)
Supplement: Supplementary file 2 — Additional file 2: Complete results of 4-taxon simulations based on amino acid data. Graphical result plots of all AliGROOVE analyses performed for amino acid data based on 4-taxon topologies. The pdf document can be opened with pdf readers like AdobeAcrobatReader, Xpdf, or DocumentViewer. (PDF 65 KB) [file 12859_2013_6580_MOESM2_ESM.pdf]

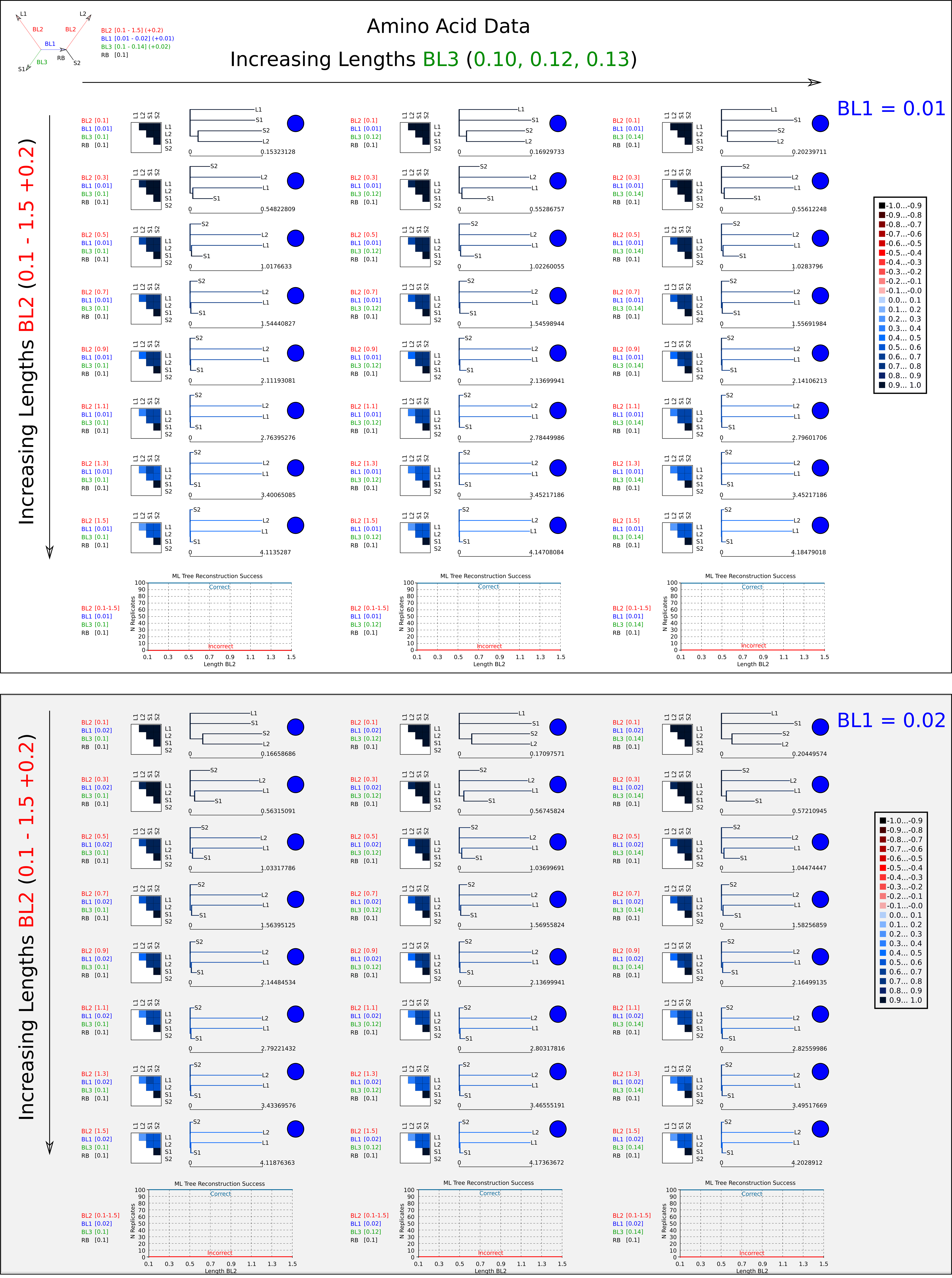

AliGROOVE additional result file A2: 4-taxon simulation studies (amino acid data, sequence lengths: 250,000 bp). Correctly aligned test data have been generated for a 4-taxon topology under 48 different branch length conditions (two terminal branch lengths BL2 stepwise increased from 0.1 to 1.5 in steps of 0.2 under three stepwise elongated lengths of a third terminal branch BL3=0.10, 0.12, 0.14 and a constant length of the remaining terminal branch RB=0.1 while internal branch length BL1 has been set either to 0.01 (above) or 0.02 (below, shaded grey)) using the BLOSUM62 amino acid substitution model with an α-shape parameter of rate heterogeneity α=1.0, a continuous Γ-distribution, and an invariant proportion of 0.3. Maximum Likelihood (ML) analyses have been performed under identic model parameter settings, except of using four discrete α-shape categories than a continuous Γ-distribution for tree estimates. AliGROOVE similarity score distance matrices and associated ML topologies are given for the first of 100 data replicates generated for each branch length condition. The darker blue the colour coded similarity scores in AliGROOVE matrices, the higher the non-randomized accordancy between pairwise taxon sequences. Red indicates the opposite. AliGROOVE tagged branch reliability of associated best ML topologies is given next to each matrix. Correct reconstructed topologies are pointed blue, incorrect trees red. The overall ML reconstruction success observed for 100 data replicates of each branch length condition are shown for each single lengths of BL1 and BL3.
