# Supplementary material for: AliGROOVE – visualization of heterogeneous sequence divergence within multiple sequence alignments and detection of inflated branch support
Source: BMC Bioinformatics. 2014 Aug 30;15(1):294. doi: 10.1186/1471-2105-15-294 (PMC4167143; doi:10.1186/1471-2105-15-294)
Supplement: Supplementary file 3 — Additional file 3: Complete results of 6-taxon simulations. Graphical result plots of all AliGROOVE analyses performed for nucleotide and amino acid data based on 6-taxon topologies. The pdf document can be opened with pdf readers like AdobeAcrobatReader, Xpdf, or DocumentViewer. (PDF 31 KB) [file 12859_2013_6580_MOESM3_ESM.pdf]

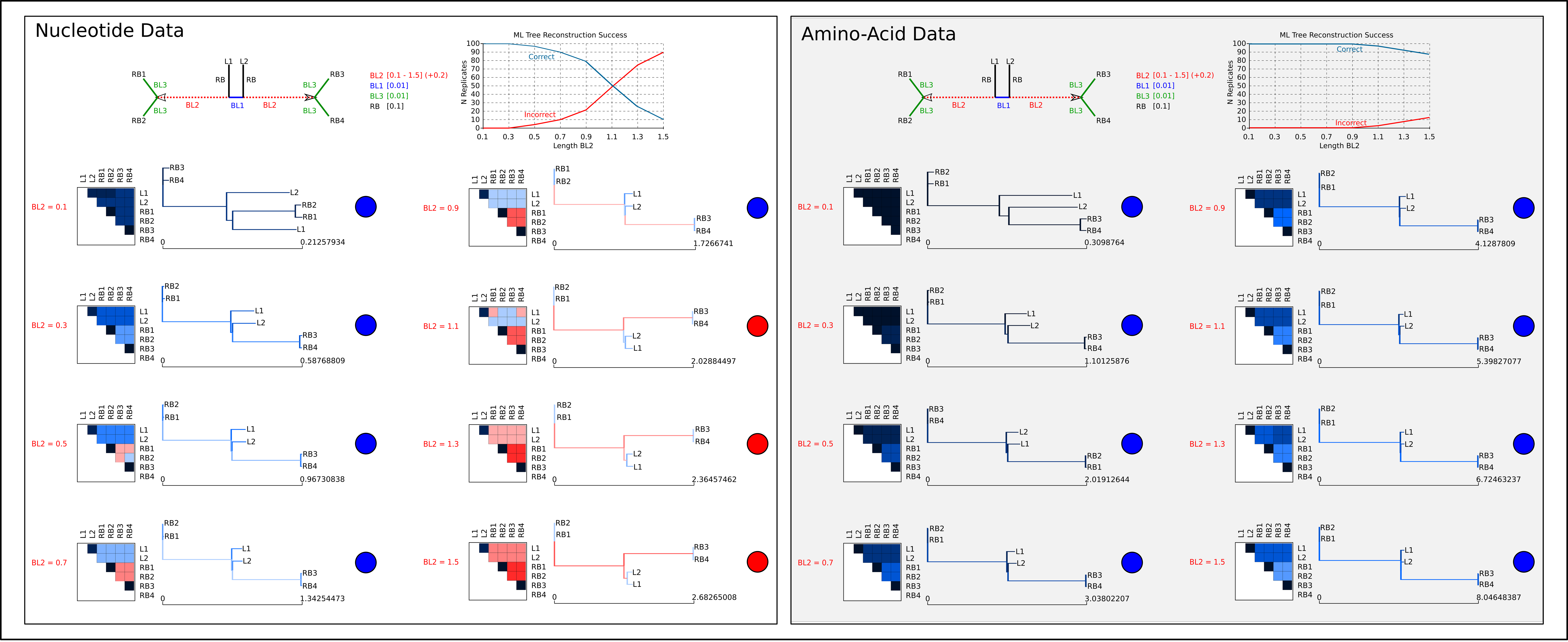

AliGROOVE additional result file A3: 6-taxon simulation studies (nucleotide data (left) and amino-acid data (right, shaded grey), sequence lengths: 50,000 bp). Correctly aligned test data have been generated for a 6-taxon topology under eight different branch length conditions (internal branch lengths BL2 stepwise increaesed from 0.1 to 1.5 in steps of 0.2 while terminal branch lengths have been kept constant) using the Jukes-Cantor (JC) model of sequence evolution (nucleotide data) and the BLOSUM 62 substitution matrix (amino-acid data) with an α-shape parameter of rate heterogeneity α=1.0, a continuous Γ-distribution, and an invariant proportion of 0.3. Maximum Likelihood (ML) analyses have been performed under identic model parameter settings, except of using four discrete α-shape categories than a continuous Γ-distribution for tree estimates. AliGROOVE similarity score distance matrices and associated ML topologies are given for the first of 100 data replicates generated for each branch length condition. The darker blue the colour coded similarity scores in AliGROOVE matrices, the higher the non-randomized accordancy between pairwise taxon sequences. Red indicates the opposite. AliGROOVE tagged branch reliability of associated best ML topologies is given next to each matrix. Correct reconstructed topologies are pointed blue, incorrect trees red. The overall ML reconstruction success observed for 100 data replicates of each branch length condition are shown for nucleotide and amino-acid data.
