# Supplementary material for: AliGROOVE – visualization of heterogeneous sequence divergence within multiple sequence alignments and detection of inflated branch support
Source: BMC Bioinformatics. 2014 Aug 30;15(1):294. doi: 10.1186/1471-2105-15-294 (PMC4167143; doi:10.1186/1471-2105-15-294)
Supplement: Supplementary file 8 — Additional file 8: Complete results of taxon reduced gene partitions based on 61-taxon amino acid simulations. Graphical result plots of all AliGROOVE analyses performed for taxon reduced amino acid partitions based on 61-taxon topologies. The pdf document can be opened with pdf readers like AdobeAcrobatReader, Xpdf, or DocumentViewer. (PDF 288 KB) [file 12859_2013_6580_MOESM8_ESM.pdf]

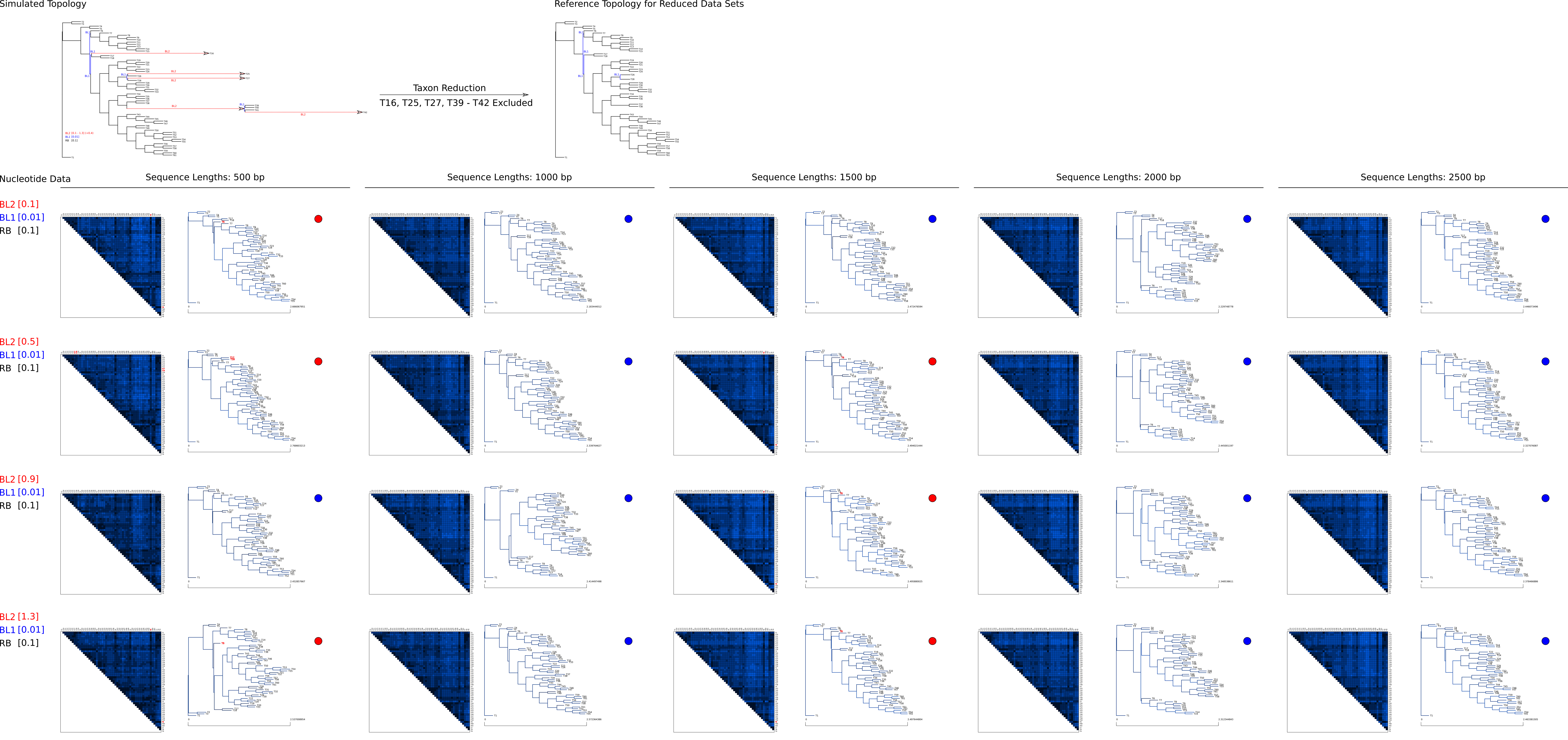

AliGROOVE additional result file A8: 61-taxon simulation studies (nucleotide data, sequence lengths: 500 - 2500 bp). Correctly aligned test data have been generated for a 61-taxon topology under different branch length conditions (BL2 stepwise increased from 0.1 to 1.3 in steps of 0.4 under constant length of the remaining branches RB=0.1 while branch length BL1 has been set to 0.01) using the Jukes-Cantor (JC) model of sequence evolution with an α-shape parameter of rate heterogeneity α=1.0, a continuous Γ-distribution, and an invariant proportion of 0.3. Maximum Likelihood (ML) analyses have been performed under identic model parameter settings, except of using four discrete α-shape categories than a continuous Γ-distribution for tree estimates. AliGROOVE similarity score distance matrices and associated ML topologies are given for each branch length condition. The darker blue the colour coded similarity scores in AliGROOVE matrices, the higher the non-randomized accordancy between pairwise taxon sequences. Red indicates the opposite. AliGROOVE tagged branch reliability of associated best ML topologies is given next to each matrix. Correct reconstructed topologies are pointed blue, incorrect trees red.
