# Supplementary material for: AliGROOVE – visualization of heterogeneous sequence divergence within multiple sequence alignments and detection of inflated branch support
Source: BMC Bioinformatics. 2014 Aug 30;15(1):294. doi: 10.1186/1471-2105-15-294 (PMC4167143; doi:10.1186/1471-2105-15-294)
Supplement: Supplementary file 4 — Additional file 4: Complete results of 15-taxon simulations. Graphical result plots of all AliGROOVE analyses performed for complete and indel events included nucleotide data based on 15-taxon topologies. The pdf document can be opened with pdf readers like AdobeAcrobatReader, Xpdf, or DocumentViewer. (PDF 251 KB) [file 12859_2013_6580_MOESM4_ESM.pdf]

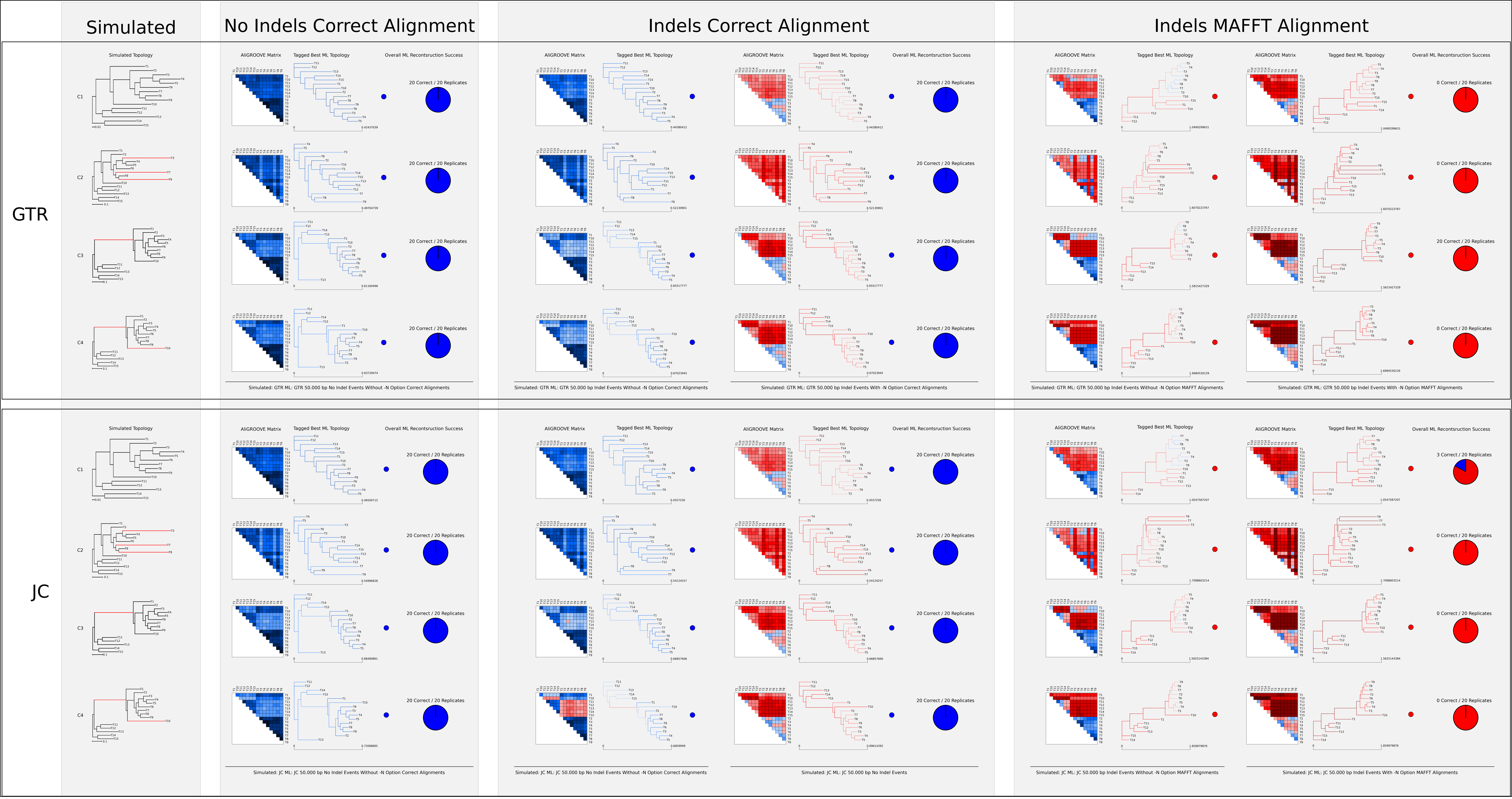

AliGROOVE additional result file A4: 15-taxon simulation studies (nucleotide data, sequence lengths: 50,000 bp). Correctly aligned and unaligned test data have been generated for a 15-taxon topology under four different branch length conditions and two evolutionary models of base substitutions (Jukes-Cantor (JC) and GTR) with an α-shape parameter of rate heterogeneity α=0.5 , a continuous Γ-distribution, and an invariant proportion of 0.1. Maximum Likelihood (ML) analyses have been performed simulated model parameters for correctly aligned data and estimated model parameters for MAFFT aligned data under otherwise equal conditions, except of using four discrete α-shape categories than a continuous Γ-distribution for tree estimates. AliGROOVE similarity score distance matrices and associated ML topology are given for the first of 20 data replicates generated for each branch length conditions. The darker blue the colour coded similarity scores in AliGROOVE matrices, the higher the non-randomized accordancy between pairwise taxon sequences. Red indicates the opposite. AliGROOVE tagged branch reliability of associated best ML topologies is given next to each matrix. Correct reconstructed topologies are pointed blue, incorrect trees red. The overall ML reconstruction success observed for twenty data replicates of each branch length condition are shown next to each single set of AliGROOVE results.
